# Simplified quantification method for in vivo SPECT/CT imaging of asialoglycoprotein receptor with ^99m^Tc-*p*(VLA-co-VNI) to assess and stage hepatic fibrosis in mice

**DOI:** 10.1038/srep25377

**Published:** 2016-05-06

**Authors:** Deliang Zhang, Zhide Guo, Pu Zhang, Yesen Li, Xinhui Su, Linyi You, Mengna Gao, Chang Liu, Hua Wu, Xianzhong Zhang

**Affiliations:** 1 Center for Molecular Imaging and Translational Medicine, State Key Laboratory of Molecular Vaccinology and Molecular Diagnostics, School of Public Health, Xiamen University, Xiamen 361102, China; 2 Department of Isotope, China Institute of Atomic Energy, P. O. Box 2108, Beijing 102413, PR China; 3 Department of Nuclear Medicine, The First Affiliated Hospital of Xiamen University, Xiamen 361003, China; 4 Department of Nuclear Medicine, Zhongshan Hospital affiliated to Xiamen University, Xiamen 361004, Fujian, China

## Abstract

The goal of this study is to develop a noninvasive method of SPECT imaging to quantify and stage liver fibrosis with an Asialoglycoprotein receptor (ASGP-R) targeting tracer—^99m^Tc-*p*(VLA-co-VNI). ASGP-Rs are well known to specifically express in the mammalian liver. Here, we demonstrated ASGP-R expression decreased in carbon tetrachloride (CCl_4_)-induced mouse model. ASGP-R expression correlated with liver fibrosis progression. ASGP-R could be a useful marker in the stage of liver fibrosis. Liver uptake value (LUV) derived by SPECT imaging was used to assess liver fibrosis in the CCl_4_-induced mouse model. LUV = [radioactivity (liver uptake)/radioactivity (injected)] × 100/liver volume. The LUV decreased along with the disease progression. The relationships between LUV and liver hydroxyproline (i.e. collagen), as well as Sirius Red were established and verified. A strong negative linear correlation was found between LUV and hydroxyproline levels (r = −0.83) as well as LUV and Sirius Red quantification (r = −0.83). In conclusion, SPECT imaging with ^99m^Tc-*p*(VLA-co-VNI) is useful in evaluating and staging liver fibrosis *in vivo*.

Liver fibrosis is the result of repeated liver damage which causes the sustaining activation of tissue repair mechanisms to substitute extracellular matrix (ECM) for necrotic tissue^1^. In the developed countries, alcohol abuse, chronic HCV infection, and nonalcoholic steatohepatitis (NASH) are the main causes of liver fibrosis. Historically, hepatic fibrosis was thought to be a non-reversible process due to the collapse of the hepatic parenchyma and replacement by a collagenous scar^2^. Currently, it is considered a reversible compensatory measure response to chronic hepatic injury. However, if it is left unchecked and doesn’t execute positive intervention, fibrosis will progress to cirrhosis. The major clinical consequences of cirrhosis are damage of liver function and development to hepatocellular carcinoma (HCC), both of which is associated with short survival. Moreover, morbidity and mortality are high for patients with severe liver fibrosis operating liver resection^3^,^4^.

Liver biopsy is considered as the gold-standard method for the assessment of liver fibrosis despite several limitations^5^. Histological examination is useful in identifying the leading cause of liver disease and the stage of fibrosis. However, liver biopsy is an invasive procedure with pain. Complications occur in 0.5% of patients, with mortality rates of 5.1%^6^,^7^. In addition, histological examination does not predict progression of fibrosis. More remarkable, fibrosis progression before and after transplantation has prognostic significance: serious fibrosis and early cirrhosis are associated with poor survival in patient^8^,^9^. Lack of clinical trials is because it requires long-term follow-studies to confirm patients’ condition. However, liver biopsy, an invasive procedure, is still the gold-standard method for identifying the stage of liver fibrosis. Reiterant biopsies to evaluate fibrosis progression or response to treatment are unprepossessing, due to the patient compliance and increased risk of complications. For all of these reasons, there is an urgent need to develop non-invasive ways, which can repeatedly diagnosis of chronic liver disease and monitor fibrosis progression throughout the whole organ, and this will facilitate the design of clinical trials. Unfortunately, mild to moderate stages of liver fibrosis were unable to be detected by conventional imaging although the development of elastography has shown promise in recent years^10^.

Asialoglycoprotein receptors (ASGP-Rs) which are located on the surface of hepatocytes membrane, are well known to specifically express in the mammalian liver and played a major role in the hepatic metabolism of serum proteins^11^. For patients with chronic liver disease, ASGP-Rs decrease sharply in liver^12^,^13^. Quantitative imaging of ASGP-Rs can assess the function of the liver and provided a noninvasive way to diagnose disease. The ASGP-R imaging agent can assess the anatomy and function of the liver and help the early diagnosis of hepatic diseases and accurate evaluation of functional status^14^,^15^. ^99m^Tc-GSA which is an albumin ramification has been used as an ASGP-R-binding radiopharmaceutical in clinical research in Japan since 1992^16^,^17^,^18^. In 2011, Yang *et al.* developed a new synthetic copolymer-*p*(VLA-co-VNI) with galactose-carrying monomer on the skeleton of styrene and incorporating hydrazine-nicotinamide as functionalized groups for ^99m^Tc labeling as potential ASGP-R imaging agent^19^. In 2014, Liu *et al.* synthesized copolymer ^99m^Tc-*p*(VLA-co-VNI)(46:54) with different ratio of targeting unit (galactose-carrying monomer VLA) and labeling unit (HYNIC-carrying monomer VNI) copolymer with better hepatic targeting and biological properties^20^. Here, we proved that *in vivo* SPECT/CT imaging of ASGP-Rs with ^99m^Tc-*p*(VLA-co-VNI) can reliably distinguish among different stages of liver fibrosis in the CCl_4_-induced liver fibrosis mouse model and establish a simplified quantification method to assess hepatic fibrosis progression.

## Results

### Histological characterization of the fibrotic liver in the CCl_4_-induced mouse model

**Figure 1:**
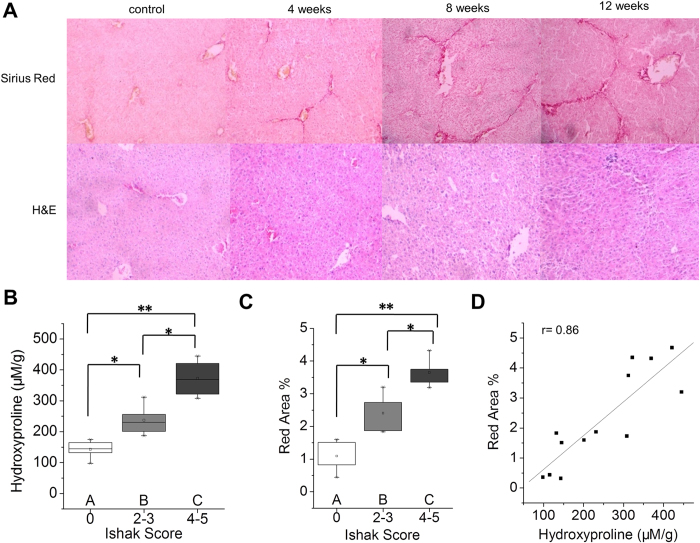
Characterization of the CCl_4_-induced fibrotic mice. (**A**) Representative images of Sirius Red and H&E staining after CCl_4_ treatment for 4, 8 or 12 weeks. (**B**) Total collagen was assessed by hydroxyproline analysis and compared to disease progression as determined by Ishak scoring. (**C**) Sirius Red staining was quantified using Image-pro-pus software and compared to disease progression. (**D**) Correlation analysis between total collagen (hydroxyproline) and Sirius Red quantification. *p < 0.05 and **p < 0.01.

For the purpose of identifying whether ^99m^Tc-*p*(VLA-co-VNI) could distinguish early stages of liver fibrosis from more advanced stages, we treated mice with CCl_4_ or olive oil (as vehicle control) for 4, 8 or 12 weeks to induce varying stages of liver fibrosis. Liver fibrosis progressed in a stepwise fashion in the CCl_4_ mouse model, as was expected and verified by Sirius Red and H&E staining (Fig. 1A). After 4 weeks of CCl_4_ administration, most mice had fibrous portal expansion with short fibrous septa (Ishak 2–3), and the disease progressed to complete bridging fibrosis with appearance of a few of regenerative nodules (Ishak 4–5) by 8–12 weeks. Consistently, we detected collagen of liver tissue by hydroxyproline analysis and collagen deposition increased along with the Ishak score (Fig. 1B). We also quantified the amount of collagen deposition by calculating the total amount of Sirius Red staining in representative slides from each animal and this measurement correlated well with Ishak score (Fig. 1C) and liver hydroxyproline levels (Fig. 1D).

### ASGP-R expression decreased in CCl_4_-induced mouse model of liver fibrosis

**Figure 2:**
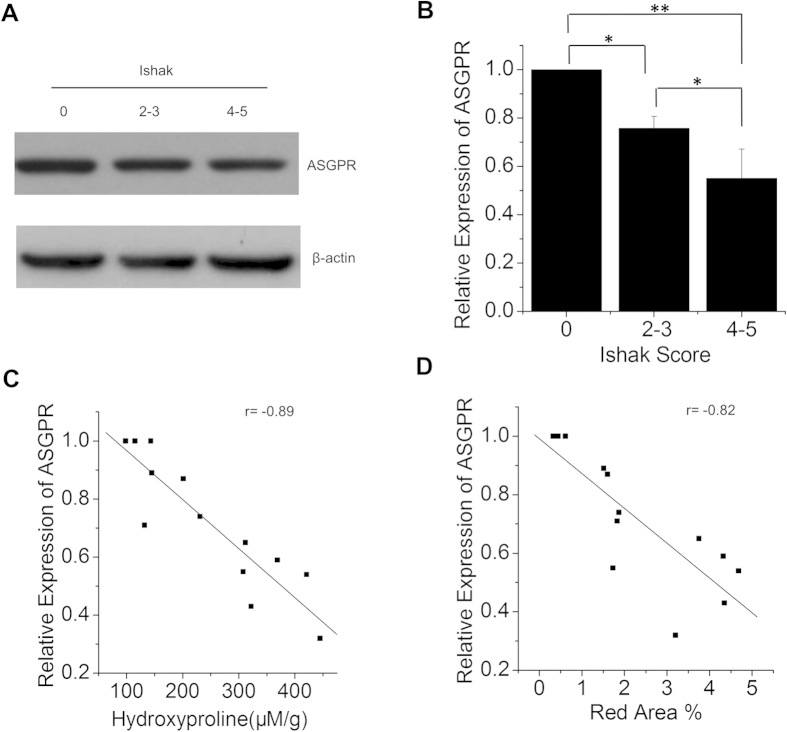
ASGP-R expression decreased in CCl_4_-induced mouse model of liver fibrosis. (**A**) Expression levels of ASGP-R determined by Western Blot in control and fibrotic mice. (**B**) Relative quantification of ASGP-R expression of CCl_4_-induced mouse model by gray scanning analysis. (**C**) Correlation between ASGP-R expression and total collagen. (**D**) Correlation between ASGP-R expression and Sirius Red quantification. *p < 0.05 and **p < 0.01.

To determine whether chronic liver injure could down-regulate ASGP-R expression in the CCl_4_-induced mouse model, we extracted proteins from the liver tissue of control and fibrotic mice to assess the expression of ASGP-R. Western bolt analysis indicated that the protein level of ASGP-R was down-regulated in the liver of fibrotic mice treated with CCl_4_ (Fig. 2A,B). Down-regulation of ASGP-R expression in the fibrotic mice was further confirmed by immunohistochemical experiment (Supplementary Fig. S1). We observed strong correlations between ASGP-R expression and total collagen levels (r = 0.89, Fig. 2C) as well as ASGP-R expression and Sirius Red quantification (r = 0.82, Fig. 2D). These results indicated that ASGP-R could act as a potential molecular marker in staging of liver fibrosis.

### SPECT/CT imaging evaluated the stage of liver fibrosis in a CCl_4_-induced liver fibrosis mouse model

**Figure 3:**
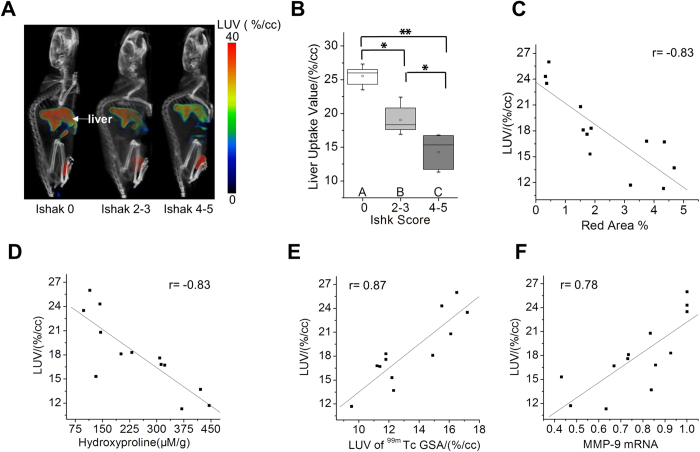
SPECT imaging evaluate the stage of liver fibrosis in CCl_4_-induced mouse model. (**A**) SPECT/CT images of control and fibrotic mice show the liver imaging. Both images were rendered at the same scale. (**B**) Liver Uptake Value (LUV) was calculated as disease progressed as determined by Ishak scoring. (**C**) Correlation between LUV and total collagen (hydroxyproline). (**D**) Correlation between LUV and Sirius Red quantification. (**E**) Correlation between LUV and LUV of ^99m^Tc-GSA. (**F**) Correlation between LUV and MMP-9 mRNA relative expression. *p < 0.05 and **p < 0.01.

^99^ ^m^Tc-*p*(VLA-co-VNI) SPECT/CT imaging was used to quantify asialoglycoprotein receptor expression and assess hepatic fibrosis in mice. LUV derived from SPECT/CT imaging was used as a simplified quantification index to quantify asialoglycoprotein receptor expression and assess the stage of liver fibrosis. LUV was calculated by dividing the liver ROI by the total inject radioactivity and liver volume. The time-activity curve of LUV derived from the dynamic SPECT imaging (Supplementary Fig. S2) showed that the liver uptake in the C57BL/6 mice peaked within 35–50 min after injection. So the static microSPECT scans were performed at 40 min after ^99m^Tc-*p*(VLA-co-VNI) injection, as seen in Fig. 3A. Strong contrast between the liver and non-targeted tissues or organs was obtained. SPECT/CT image showed specific and obvious high uptake in liver, except for metabolite in intestines and bladder. The specific binding of ^99m^Tc-*p*(VLA-co-VNI) has been performed previously via blocking experiment of biodistribution, liver uptake decreased significantly by blocking with excess amount of cold p(VLA-co-VNI)^20^. ^99m^Tc-*p*(VLA-co-VNI) could specifically target ASGP-R that was further confirmed by the competitive inhibition experiment (Supplementary Fig. S3). The LUV decreased with disease progression (Ishak score) in the fibrotic CCl_4_-induced mice, as shown in Fig. 3B. LUV of control (Ishak 0) and fibrotic (Ishak 2–3 and Ishak 4–5) mice was 25.5 ± 1.58, 19.0 ± 2.12, and 14.3 ± 2.41, respectively. There have strong correlations between LUV and Sirius Red quantification (r = 0.89, Fig. 3C) and between LUV and liver hydroxyproline levels (r = 0.83, Fig. 3D). We also observed a high correlation between LUV and MMP-9 mRNA relative expression (r = 0. 78, Fig. 3F). The relationship of LUV derived from SPECT imaging by using ^99m^Tc-*p*(VLA-co-VNI) and ^99m^Tc-GSA respectively was compared as well (r = 0.87, Fig. 3E). Both agents of ^99m^Tc-*p*(VLA-co-VNI) and ^99m^Tc-GSA for SPECT imaging were obtained with high RCP (>95%) (Supplementary Fig. S4).

### Compare the performance of LUV, HH15 and LHL15 in predicting severe hepatic fibrosis

**Figure 4:**
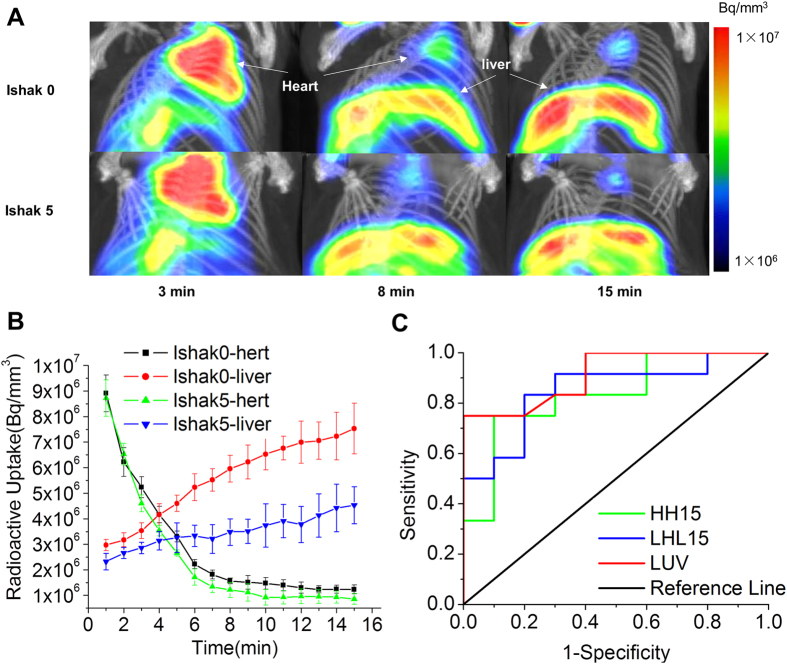
^99m^Tc-*p*(VLA-co-VNI) SPECT/CT dynamic images for predicting performance of the indices in severe fibrosis. (**A**) SPECT/CT dynamic images of control (Ishak 0) and fibrotic (Ishak 5) mice showed the liver imaging at different time. Both images rendered at the same scale. (**B**) Radioactive uptake curve of control (Ishak 0) and fibrotic (Ishak 5) mice showed the liver and heart uptake value. (**C**) ROC curves for predicting performance of the three indices in severe fibrosis.

The clearance index HH15 and the receptor index LHL15 are simple indicators used for assessment of blood clearance and hepatic functional reserve. HH15 and LHL15 were calculated by two point data points from the time-activity curves. In order to obtain the liver function indices of LHL15 and HH15, a SPECT/CT dynamic imaging were performed for 15 min with ^99m^Tc-*p*(VLA-co-VNI). Dynamic imaging of control (Ishak 0) and fibrotic (Ishak 5) mice showed the liver images at 3, 8 and 15 min, as seen in Fig. 4A. Radioactive uptake curve of control (Ishak 0) and fibrotic (Ishak 5) mice showed the liver and heart uptake value (Fig. 4B). In both of normal and fibrotic mice, liver uptake increases over time, whereas radioactivity in blood decreased rapidly. The liver uptake of fibrotic mice is much higher than that of control group and the blood clearance of fibrotic mice is faster than control group (Ishak 0 liver vs. Ishak 5 liver, P < 0.01; Ishak 0 heart vs. Ishak 5 heart, P < 0.05). We present the ROC curves for each of the 3 variables (Fig. 4C). The AUC values of LUV, LHL15 and HH15 for predicting severe liver fibrosis were 0.91, 0.88, and 0.81, respectively. LUV had significantly better performance for predicting severe fibrosis than LHL15 and HH15 (LUV vs. HH15, P < 0.01; LUV vs. LHL15, P < 0.05).

### Monitor the therapeutic efficacy of liver fibrosis by SPECT imaging after Tan IIA treatment

**Figure 5:**
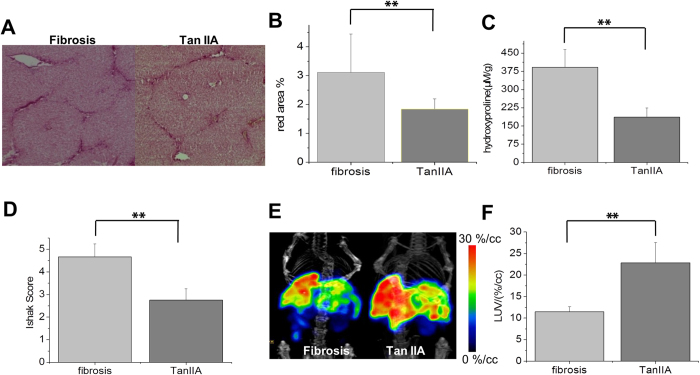
^99m^Tc-*p*(VLA-co-VNI) SPECT/CT imaging of liver fibrosis after Tan IIA treatment. (**A**) Representative images of Sirius Red staining after Tan IIA treatment quantified using Image J software (**B**) or assessed by Ishak scoring (**C**). (**D**) Total collagen was determined by hydroxyproline analysis. (**E**) SPECT/CT static images of fibrotic (Ishak 5) mice and Tan IIA treat group. (**F**) LUV was calculated. *p < 0.05 and **p < 0.01.

The lack of a non-invasive way for evaluating liver fibrosis and monitoring fibrosis progression is the main limitation in the development of anti-fibrotic drugs^21^. An assessment method based on SPECT imaging with ^99m^Tc-*p*(VLA-co-VNI) was investigated to monitor the therapeutic efficacy of liver fibrosis. Tan IIA was as potential drug for treatment of liver fibrosis, because it has multiple pharmacological activities, such as anti-oxidant^22^, anti-inflammatory^23^. As seen in Fig. 5A,B, Tan IIA had improved liver function as the extent of fibrosis assessed by Sirius Red staining. Moreover, histologic analysis of the Sirius Red staining results demonstrated that the Ishak score improved by approximately two points in the mice after Tan IIA treatment (Fig. 5C). ^99m^Tc-*p*(VLA-co-VNI) SPECT/CT static imaging showed higher liver uptake in the Tan IIA treatment group compared to the untreated group (Fig. 5E). In the Tan IIA treated group, LUV increased about twice more than control group (Fig. 5F) that correlated with the Sirius Red staining and hydroxyproline analysis (Fig. 5D). These results suggested ^99m^Tc-*p*(VLA-co-VNI) SPECT/CT imaging could be used as an important method for monitoring fibrosis progression and evaluating the effect of anti-fibrotic drugs.

## Discussion

Liver fibrosis is a reversible compensatory measure substitute necrotic hepatocytes with collagen scar. ASGP-Rs, which only express on mammalian hepatocytes, decrease in the progression of liver fibrosis^24^. We found strong negative correlations between ASGP-R expression and total collagen levels in the fibrotic liver in mice. We wanted to used SPECT/CT imaging with ^99m^Tc-*p*(VLA-co-VNI) to distinguish among different stages of liver fibrosis and proved it in the CCl_4_-induced liver fibrosis mouse model. Results obtained from planar ^99m^Tc-GSA scintigraphy are valuable for assessment of liver function in patients with liver damage^25^,^26^,^27^. However, ^99m^Tc-GSA has underlying immunogenicity and there is a risk of viral breakthrough for the blood products of HSA. Moreover, those ASGP-R imaging agents using albumin as backbone have long circulation time in blood, which is not to the advantage of imaging in liver disease. Previously, our research group developed synthetic copolymer *p*(VLA-co-VNI) as a new ASGP-R agent. *p*(VLA-co-VNI) uses PVLA as the backbone with galactose-carrying monomer on the skeleton of styrene and incorporates hydrazine-nicotinamide as functionalized groups, which can be rapid clearance from the bloodstream and specific accumulation in the liver.

Although there were many different values calculated from different dynamical models^28^,^29^, they are too complex to widely use in the clinical setting. Hepatic receptor index and blood clearance index (LHL15, HH15) are the most simply and commonly used indexes in planar dynamic scintigraphy. However, they may be not sufficient for precise assessment of hepatic functional reserve in liver diseases, because measurement of functional remaining liver volume on planar images obtained by scintigraphy is not accurate. We estimated LUV in the light of the liver uptake density reported by Sugahara^30^. As a SPECT analysis, it does not need continuous dynamic scan immediately after probe injected. This value is simple, convenient and reproducible. The combined SPECT/CT method gets accurate anatomic data on LUV, and the functional information is attenuated and scatter corrected upon CT images. As it is reported^31^,^32^,^33^, quantification of attenuated and scatter-corrected SPECT images in accordance with CT are more accurate than that based on SPECT images lack of attenuation and scatter correction. In this study, we compared the performance of HH15, LHL15, and LUV in predicting liver fibrosis. The ROC analyses showed that LUV was more accurate than LHL15 for predicting severe fibrosis. Therefore, SPECT/CT fusion images are beneficial to accurate assessment of liver fibrosis.

Here we showed for the first time that ^99m^Tc-*p*(VLA-co-VNI) SPECT/CT fusion imaging could precisely monitor and stage liver fibrosis progression in CCl_4_-treated mice. Furthermore, we observed a remarkable correlation between LUV and total collagen levels. ^99m^Tc-*p*(VLA-co-VNI) SPECT/CT imaging demonstrated LUV was reduced by 45% in the severe fibrosis mice compared to the control group. However, even in the severe fibrotic mice, the fibrotic area accounted for only 3.7% of the whole liver assessed by Sirius Red staining. Therefore, the decrease of LUV would not be as a result of substitute ECM for necrotic tissue in the fibrotic mice. Western analysis indicated that the protein level of ASGP-R was down-regulated approximate 50% in the liver of severe fibrotic mice correlated with the change of LUV. All of above results suggested LUV decrease was result from alterations of ASGP-R expression rather than collagen deposition. And then we observed a remarkable correlation between ASGP-R expression and total collagen levels. Therefore, it is viable to monitor progression of the fibrosis by quantifying ASGP-R expression and assessing hepatic functional reserve. Though histologic examination is useful in identifying the potential cause of liver diseases and evaluating the inflammatory grade and the stage of fibrosis, histological scoring suffers from the limitation of biopsy and only represents a very small sample of the whole liver. Therefore, ^99m^Tc-*p*(VLA-co-VNI) SPECT/CT imaging of the entire liver might better represent disease stage than histological scoring of biopsied liver tissue.

Further study is needed to determine whether ^99m^Tc-*p*(VLA-co-VNI) SPECT/CT imaging can be used to monitor fibrosis progression in response to treatment. Removing the causative agent is the most effective intervention in the treatment of liver fibrosis. However, in some case, it is difficult to remove the insulting agent of liver diseases (for example, HCV and fatty liver). We used Tan IIA for treatment of liver fibrosis, just because of multiple pharmacological activities of Tan IIA, such as anti-oxidant, anti-inflammatory. As it is reported, the inflammatory factors (e.g., IL-1β, IL-6, SOCS1, and IFN-γ) facilitate the activation of HSC and promote the fibrotic progression^34^,^35^,^36^ and genes mediating ROS generation (e.g., NADPH oxidase) regulate both inflammation and ECM deposition^37^. The results of our study suggest that treatment with Tan IIA can degrade collagen fibers and reduce liver fibrosis. Increased LUV were observed in Tan IIA treated group in SPECT/CT imaging with ^99m^Tc-*p*(VLA-co-VNI). Here we report the non-invasive method based on SPECT/CT imaging with ^99m^Tc-*p*(VLA-co-VNI) can be greater benefit to monitor fibrosis progression and evaluate the efficacy of anti-fibrotic drugs comprehensively.

Several radiotracers targeting other receptors were reported used for noninvasive fibrosis imaging. Muzard *et al.* described a ^99m^Tc labeled peptidomimetic of the platelet collagen receptor glycoprotein VI by targeting collagen^38^. The radiotracer was demonstrated to successfully image fibrosis *in vivo* in a mouse model of lung fibrosis. Yu *et al.* reported a αvβ3–targeting radiotracer ^99m^Tc-3PRGD2^39^. The radiotracer could identify liver fibrosis as early as 4 weeks after TAA treatment. Compared with these previous radiotracer, ^99m^Tc-p(VLA-co-VNI) showed higher uptake in liver and lower kidney accumulation which was beneficial for the quantification of liver uptake. In our study we defined a simplified quantification index LUV and conducted correlation analysis of LUV with physiological index of liver fibrosis. We demonstrated that SPECT/CT with ^99m^Tc-p(VLA-co-VNI) could reliably distinguish among different stages of liver fibrosis in the CCl_4_-induced liver fibrosis mouse model. However, ^99m^Tc-p(VLA-co-VNI) as a ASGP-R targeting radiotracer was only useful for detection of liver fibrosis rather than else fibrotic diseases.

In conclusion, ^99m^Tc-*p*(VLA-co-VNI) SPECT and CT fusion imaging is useful for accurately stage fibrosis and quantitative evaluation the functional reserve of the liver in CCl_4_-treated mice. The LUV is the most important SPECT/CT value for diagnosing histological liver damage and predicting severe fibrosis. ^99m^Tc-*p*(VLA-co-VNI) SPECT can be a valuable tool for predicting fibrosis response to therapy and selecting more appropriate treatment regimens for patients with chronic liver disease.

## Mehtods

### Animal models

The experimental procedures and the animal use and care protocols were approved by the Institutional Animal Care and Use Committee of Xiamen University. All experimental protocols were carried out in accordance with the relevant guidelines. C57BL/6 mice obtained from the Laboratory Animal Center of Xiamen University were administered 0.1 mL of a 40% solution of CCl_4_ (Sigma, St. Louis, MO) in olive oil by intraperitoneal injection, two times a week for either 4, 8 or 12 weeks, to induce fibrosis at different stages (n = 6 for all time points). Controls received only pure olive oil. Animals were imaged one week after the last injection to avoid acute effects of CCl_4_. Tanshinone II-A (Tan IIA), the main active diterpene quinone extracted from Salvia miltiorrhiza was used for the treatment of liver fibrosis; One group of mice was administered with 0.1 mL of a 40% solution of CCl_4_ while another group was administered with 100 mg/kg Tan IIA (added in a same dose CCl_4_ solution) by intraperitoneal injection, two times a week for 9 weeks (n = 6 for each group).

### Probes

The radiolabeling procedure was similar to the previously published literature^19^,^20^ Briefly, the optimal proportion of *p*(VLA-co-VNI)(46:54) lyophilized kit are 1 mg copolymer, 5 mg TPPTS, 6.5 mg Tricine, 12.7 mg succinic acid, 34.5 mg disodium succinate hexahydrate and 40 mg mannitol. For radiolabeling, the sealed kit was added 0.5 mL of freshly eluted ^99m^TcO_4_^−^ (60–100 MBq) from a commercial generator (HTA Co. Ltd., China) and heated for 20 min at 100 °C. After reaction, the crude product was passing through a 0.22 μm millipore filter to obtain the resulting complex ^99m^Tc-*p*(VLA-co-VNI)(46:54)(Tricine)(TPPTS) (shorted as ^99m^Tc-*p*(VLA-co-VNI)).

^99m^Tc-GSA was obtained by using an instant kit method (lyophilized GSA kits were donated by Beijing Shihong Pharmaceutical Center) for comparison. Briefly, by adding 0.5 mL elution of ^99m^TcO_4_^−^ (180–300 MBq) to a GSA kit, followed by incubation at room temperature for 15 min.

Both of the radiochemistry purity (RCP) of ^99m^Tc-*p*(VLA-co-VNI) and ^99m^Tc-GSA were evaluated by using ITLC/ACD chromatography system (Supplementary materials and methods 1.4).

### Western Blot Analysis

Approximately 40 μg total liver tissues’ extracts were estimated using the Bio-Rad protein assay system. Samples were resolved on a 10% SDS-polyacrylamide gel and then transferred to a polyvinylidene difluoride membrane (Millipore, Bedford, MA, USA). After blocking with 10% bovine serum albumin, the blot was incubated with anti-ASGP-R1 antibody (abcam, ab88042; 1:1,000 dilution). Horseradish peroxidase-conjugated secondary antibodies (Sigma, St. Louis, MO, USA) were typically diluted 1:5000 and chemiluminescent substrate (Amersham Pharmacia Biotech, Piscataway, NJ, USA) was used as described in the manufacturer’s instructions.

### mRNA quantification

Total RNA was extracted from liver tissues and complementary DNA was synthesized. qPCR for mRNA was performed on a real-time PCR system (CFX96, Bio-Rad, CA, USA). Primers used are listed as follows: MMP9-F: AGG ATG GGA AGT ACT GGC GAT TCT CTG; MMP9-R: ACT TGG TCC ACC TGG TTC AAC TCA CTC and beta-actin-F: GAC CTC TAT GCC AAC ACA GTG C; beta-actin-R: GTA CTC CTG CTT GCT GAT CCA C.

### *In vivo* imaging

SPECT/CT imaging studies were carried out in accordance with the approved guidelines. SPECT/CT imaging was performed using nanoScan SC (Mediso Medical Imaging System) equipped with pinhole collimator under standard animal scan procedure. The CT acquiring parameters were as follows: energy peak of 50 kV, 670 μA, 480 projections, medium zoom. SPECT acquiring parameters were as follows: energy peak of 140 keV for ^99m^Tc, window width of 20%, matrix of 256 × 256, medium zoom, and frame: 30 s. Dynamic SPECT acquiring parameters were as follows: 20 scans, and 30 sec/frame from 0–15 min.

SPECT/CT imaging of normal C57BL/6 mice and fibrotic mice under anesthesia by isoflurane to evaluate the specific binding to ASGP-R were performed with ^99m^Tc-*p*(VLA-co-VNI). The normal C57BL/6 mice were acquired under anesthesia 40 min after injecting via the tail vein with 18 MBq of ^99m^Tc-*p*(VLA-co-VNI). The static and dynamic SPECT/CT imaging of hepatic fibrosis mice (control, experiment and Tan IIA treated group) were performed for comparison, respectively.

Calculation of the dynamic data HH15 and LHL15: time-activity curves of the liver and heart were derived from the radioactivity measured in the region of interest (ROI) of the whole liver and radioactivity measured in the ROI of the heart^40^; the blood clearance index (HH15) was calculated by dividing the radioactivity measured in ROI of the heart at 15 min after injection by the radioactivity measured in ROI of the heart at 3 min^41^. The receptor index (LHL15) was calculated by dividing the radioactivity of ROI of the liver by the sum of the radioactivity of the liver and heart ROIs at 15 min after injection^41^−^43^.

In order to further validate the feasibility of this new developed methodology, SPECT/CT imaging of normal C57BL/6 mice and hepatic fibrosis mice with known agent ^99m^Tc-GSA were also performed for comparison.

### Tissue analysis

Paraformaldehyde-fixed samples were embedded in paraffin, cut into 5 μm-thick sections and stained with Sirius Red according to standard procedures. Sirius Red stained sections were analyzed by three pathologists, who were blinded to the study. Fibrosis stage is assessed by using scales of Ishak score. Hydroxyproline in liver tissue was quantified by HPLC analysis according to previous reports^44^.

### Statistics

Results are expressed as mean ± standard error of the mean. Differences among groups were evaluated by one way analysis of variance (ANOVA) followed by a Newman-Keuls post hoc test. The Pearson correlation coefficient was computed to assess quality of linear correlations and significance was evaluated by Two-tailed test. Receiver operating curve (ROC) analysis was performed using Origin 8. Differences were considered significant if *P* < 0.05.

## Additional Information

**How to cite this article**: Zhang, D. *et al.* Simplified quantification method for in vivo SPECT/CT imaging of asialoglycoprotein receptor with ^99m^Tc-*p*(VLA-co-VNI) to assess and stage hepatic fibrosis in mice. *Sci. Rep.* **6**, 25377; doi: 10.1038/srep25377 (2016).

## Supplementary Material

Supplementary Information
