# Supplementary material for: Simplified quantification method for in vivo SPECT/CT imaging of asialoglycoprotein receptor with 99mTc-p(VLA-co-VNI) to assess and stage hepatic fibrosis in mice
Source: Sci Rep. 2016 May 6;6:25377. doi: 10.1038/srep25377 (PMC4858650; doi:10.1038/srep25377)
Supplement: Supplementary Information [file srep25377-s1.doc]

**Simplified quantification method for in vivo SPECT/CT imaging of asialoglycoprotein receptor with ^99m^Tc-*p*(VLA-co-VNI) to assess and stage hepatic fibrosis in mice**

Deliang Zhang^1^, Zhide Guo^1,2^, Pu zhang^1^,Yesen Li^1,3^, Xinhui Su^4^, Linyi You^1^, Mengna Gao^1^, Chang Liu^1^, Hua Wu^3^, Xianzhong Zhang^1^*

*^1^Center for Molecular Imaging and Translational Medicine, State Key Laboratory of Molecular Vaccinology and Molecular Diagnostics, School of Public Health, Xiamen University, Xiamen361102, China*

*^2^Department of Isotope, China Institute of Atomic Energy, P. O. Box 2108, Beijing 102413, PR China*

*^3^Department of Nuclear Medicine, The First Affiliated Hospital of Xiamen University, Xiamen 361003, China*

*^4^Department of Nuclear Medicine, Zhongshan Hospital affiliated to Xiamen University, Xiamen 361004, Fujian, China*

* For correspondence contact:

Xianzhong Zhang, Professor, PhD

School of Public Health, Xiamen University

Xiang’an South Rd., Xiang’an district, Xiamen 361102, China.

Phone: +86(592)2880645; Fax: +86(592)2880645

E-mail: zhangxzh@xmu.edu.cn

This file includes:

1. Materials and methods
2. Supplementary Fig S1-S4

**SUPPLEMENTAL INFORMATION**

1. **Materials and methods**

**1.1 Cell culture**

LO2 cells were cultured in 1640 Medium (GIBCO, Carlsbad, CA, USA) supplemented with 10 % (v/v) fetal bovine serum (FBS) at 37 °C in a humidified atmosphere with 5 % CO_2_.

**1.2 Histopathology and immunohistochemistry**

IHC was performed using anti-ASGP-R1 antibody (Abcam, ab88042; 1:200 dilution). Briefly, paraformaldehyde-fixed samples were embedded in paraffin, cut into 5 μm-thick sections, rinsed with PBS and blocked with 10 % BSA for 30 min at room temperature. The slices were incubated with anti-ASGP-R1 antibody for 1 h at room temperature and then visualized with biotin-avidin-horseradish peroxidase kit (Maixin-Bio, China) used as described in the manufacturer’s instructions.

**1.3 In vitro receptor-binding studies**

In vitro ASGP-R-binding affinities and specificities of the *p*(VLA-co-VNI) were assessed via displacement cell-binding assays using ^99m^Tc-*p*(VLA-co-VNI), as the ASGP-R-specific radioligand. LO2 Cells harvested and seeded in 24-well plates at 10^5^ cells per well. Twenty-four hours later, the cells were washed twice with binding buffer containing 50 mmol/L *N*-(2-hydroxyethyl)piperazine-*N’*-(2-ethanesulfonic acid, 125 mmol/L NaCl, 7.5 mmol/L KCl, 5.5 mmol/L MgCl2, 1 mmol/L ethylene glycol-bis-(β-aminoethylester)-*N,N,N,N*-tetraacetic acid, 2 mg/mL bovine serum albumin, 2 mg/L chymostatin, 100 mg/L soybean trypsin inhibitor at pH 7.4 and then incubated for 1 h at 37 °C with 20,000 cpm of ^99m^Tc-*p*(VLA-co-VNI) in the presence of increasing concentrations of p(VLA-co-VNI) or GSA ranging from 0 to 2,000 nmol/L. After incubation, the cells were washed twice with binding buffer and solubilized with 1N NaOH, and activity was measured in γ-counter (Packard). The 50 % inhibitory concentration (IC_50_) value for the displacement binding of ^99m^Tc-*p*(VLA-co-VNI) by p(VLA-co-VNI) or GSA was calculated by nonlinear regression analysis using the GraphPad Prism computer-fitting program (GraphPad Software, Inc.).

**1.4 The radiochemistry purity of ^99m^Tc-*p*(VLA-co-VNI) and ^99m^Tc-GSA**

The radiochemistry purities (RCPs) of ^99m^Tc-*p*(VLA-co-VNI) and ^99m^Tc-GSA were calculated by using ITLC-SG chromatography with acid-citrate-dextrose buffer (ACD, citrate [0.068 mol/L], glucose [0.074 mol/L], pH=5.0) as mobile phase. ^99m^Tc-p(VLA-co-VNI) and ^99m^Tc-GSA were remained at the point of spotting (R_f_ = 0–0.1), while other radioactive impurities moved with the solvent front (R*f* = 0.8–1.0).

1. **Supplementary Fig S1-S4**


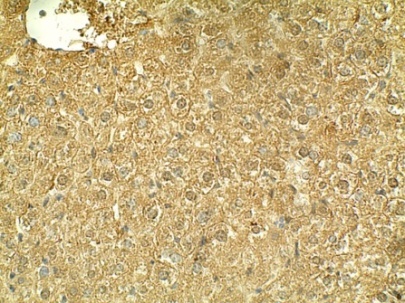

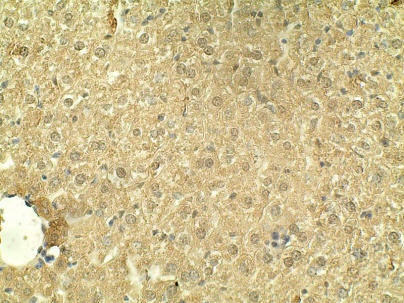


B

A

**Supplementary Fig. S1. Histological sections of the normal and fibrotic murine liver.** Histopathology and immunohistochemistry show the ASGP-R expression in the liver of normal **(A)** and fibrotic **(B)** mice.

**Supplementary Fig. S2.** The time-activity curves of LUV derived from the dynamic SPECT imaging of normal C57BL/6 mice.

**A B**


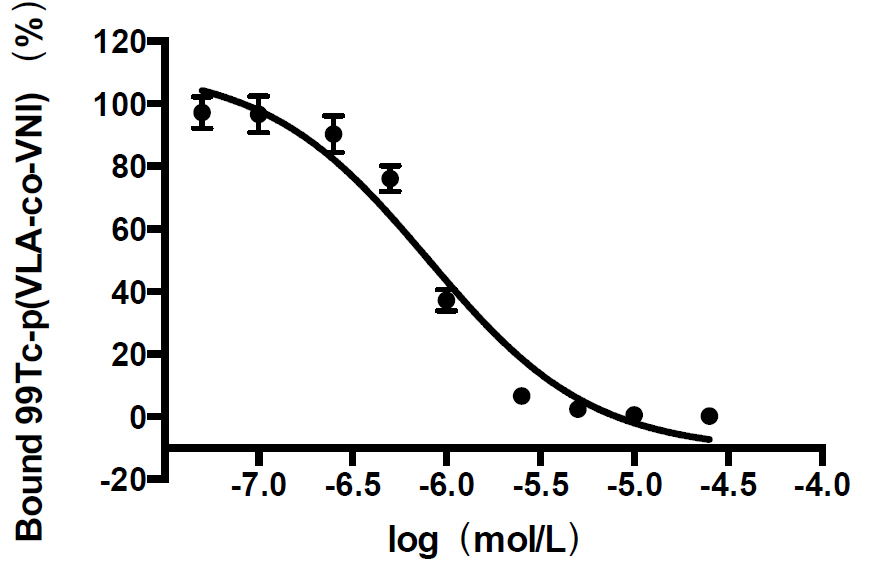

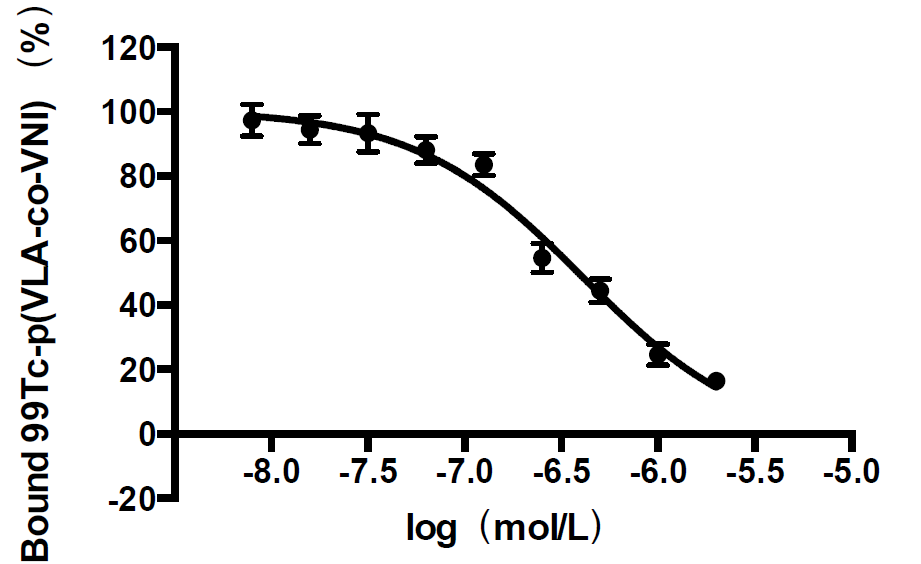


**Supplementary Fig. S3. Effect of cold ligands (untagged ligands) on the binging of** **^99m^Tc-*p*(VLA-co-VNI) by LO2 cells. (A)** Inhibition of ^99m^Tc-*p*(VLA-co-VNI) binding to ASGR-R on human liver cell line LO2 by p(VLA-co-VNI) (IC_50_= 802 ± 72.4 nM). **(B)** Inhibition of ^99m^Tc-*p*(VLA-co-VNI) binding to ASGR-R on human liver cell line LO2 by GSA (IC_50_= 406 ± 59.1 nM)

**A B**


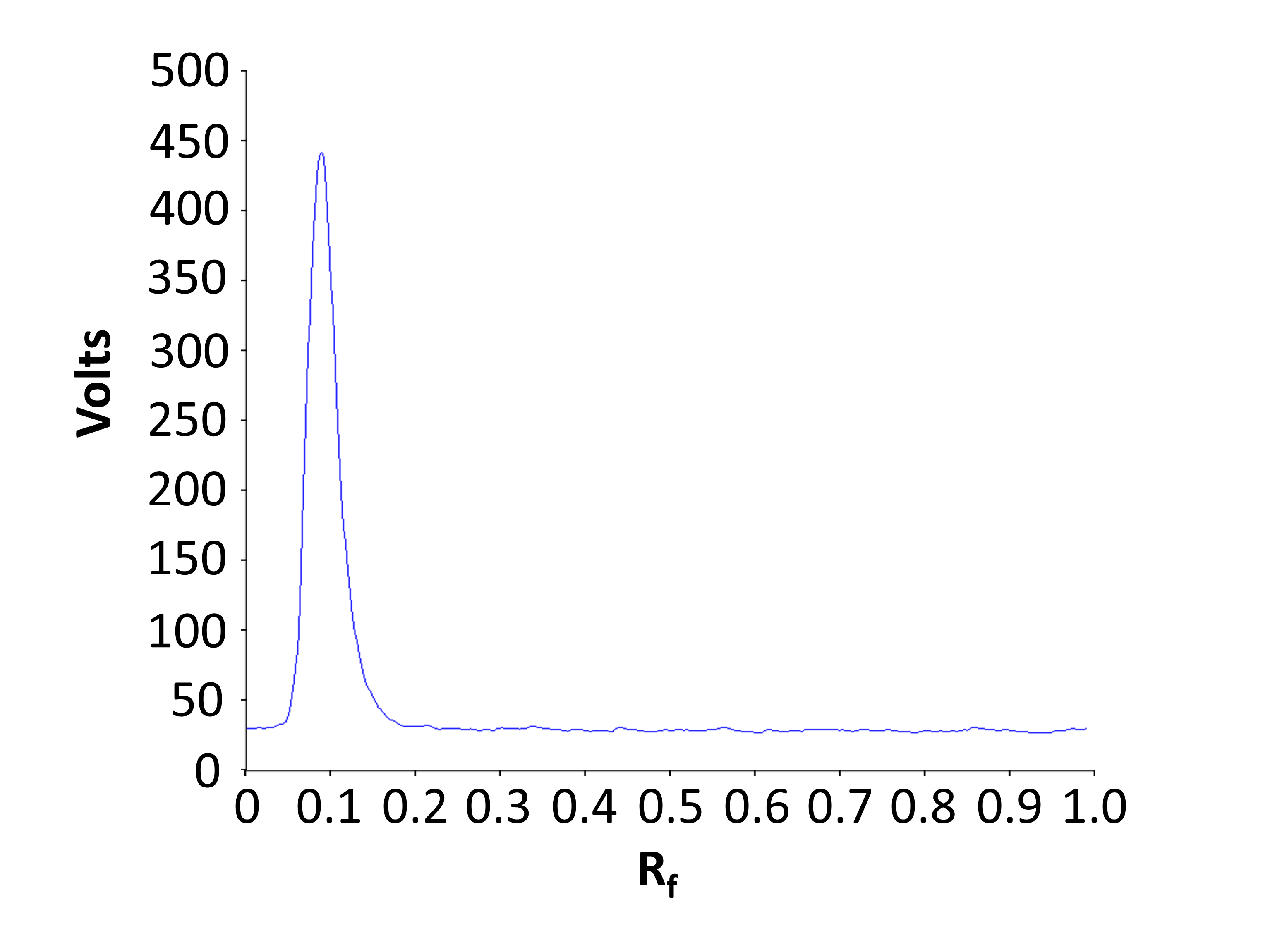

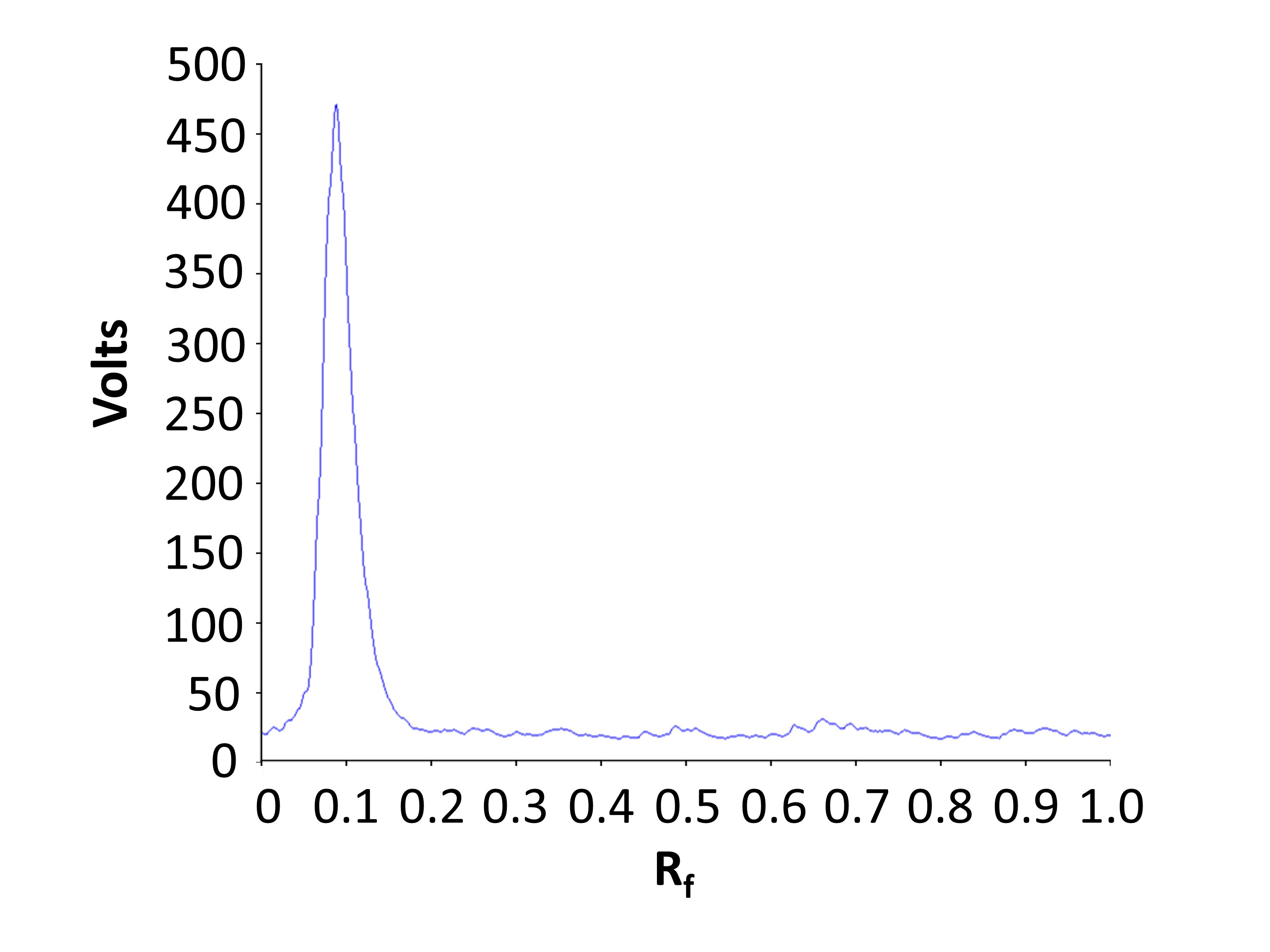


^99m^Tc-p(VLA-co-VNI)

^99m^Tc-GSA

**Supplementary Fig. S4. The TLC results ^99m^Tc-*p*(VLA-co-VNI) and ^99m^Tc-GSA identified by MiniSCAN.** ^99m^Tc-*p*(VLA-co-VNI) and ^99m^Tc-GSA were remained at the point of spotting (R_f_ = 0–0.1), while other radioactive impurities moved with the solvent front (R*f* = 0.8–1.0). Both agents of ^99m^Tc-*p*(VLA-co-VNI) and ^99m^Tc-GSA were obtained with high RCP (> 95%).
